# Detection of COVID-19 Virus on Surfaces Using Photonics: Challenges and Perspectives

**DOI:** 10.3390/diagnostics11061119

**Published:** 2021-06-19

**Authors:** Bakr Ahmed Taha, Yousif Al Mashhadany, Nur Nadia Bachok, Ahmad Ashrif A Bakar, Mohd Hadri Hafiz Mokhtar, Mohd Saiful Dzulkefly Bin Zan, Norhana Arsad

**Affiliations:** 1 UKM—Department of Electrical, Electronic and Systems Engineering, Faculty of Engineering and Built Environment, Universiti Kebangsaan Malaysia, UKM Bangi 43600, Malaysia; p103537@siswa.ukm.edu.my (B.A.T.); p110441@siswa.ukm.edu.my (N.N.B.); ashrif@ukm.edu.my (A.A.A.B.); hadri@ukm.edu.my (M.H.H.M.); saifuldzul@ukm.edu.my (M.S.D.B.Z.); 2 Department of Electrical Engineering, College of Engineering, University of Anbar, Anbar 00964, Iraq; yousif.mohammed@uoanbar.edu.iq

**Keywords:** photonics, COVID-19, laser diagnosis techniques, optical biosensor, coronavirus, light–matter interaction

## Abstract

The propagation of viruses has become a global threat as proven through the coronavirus disease (COVID-19) pandemic. Therefore, the quick detection of viral diseases and infections could be necessary. This study aims to develop a framework for virus diagnoses based on integrating photonics technology with artificial intelligence to enhance healthcare in public areas, marketplaces, hospitals, and airfields due to the distinct spectral signatures from lasers’ effectiveness in the classification and monitoring of viruses. However, providing insights into the technical aspect also helps researchers identify the possibilities and difficulties in this field. The contents of this study were collected from six authoritative databases: Web of Science, IEEE Xplore, Science Direct, Scopus, PubMed Central, and Google Scholar. This review includes an analysis and summary of laser techniques to diagnose COVID-19 such as fluorescence methods, surface-enhanced Raman scattering, surface plasmon resonance, and integration of Raman scattering with SPR techniques. Finally, we select the best strategies that could potentially be the most effective methods of reducing epidemic spreading and improving healthcare in the environment.

## 1. Introduction

The sudden and rapid emergence of severe acute respiratory syndrome coronavirus 2 (SARS-COV-2) caused the novel coronavirus disease (COVID-19). According to the Center for Systems Science and Engineering at Johns Hopkins University, there are, to date, 171,680,288 cases of COVID-19, including 3,691,680 deaths worldwide [1]. Growing global business and travel are considered the cause of frequent and rapid propagation of infectious diseases worldwide. Correspondingly, faster and on-site diagnosis decisions have also contributed to reducing the spreading of the virus and pandemic disease transmission [2,3], which has a 2–7-day incubation duration before infection initiation. This period is primarily asymptomatic and contagious, as the virus spreads from infected to healthy individuals [4].

Photonics techniques are the science of energy generating, detecting, and transmitting information using light, applying it in every part of life, from microscopy to optical communications. Likewise, it includes cutting-edge applications of lasers, optics, fiber optics, and electro-optical systems in a wide variety of areas of technology, including engineering, healthcare, telecommunications, environmental control, national security, aerospace, and solid-state illumination. As a result, this technique has opened up new challenges in different fields. Many solutions have been discovered and created by optical engineering in photonic systems when faced with global pandemic threats [5,6,7,8]. Therefore, due to its rapidity and precision, detection techniques based on photonics have appeared as a viable alternative to traditional methods or immunoassay-based methods for rapidly diagnosing viruses and epidemic diseases. Thus, photonics researchers and companies have made significant contributions to diagnostics and personal protection equipment through integrating advanced systems and creating revolutionary developments in this area [9,10,11,12].

### 1.1. Emergence and Features of COVID-19

**Figure 1:**
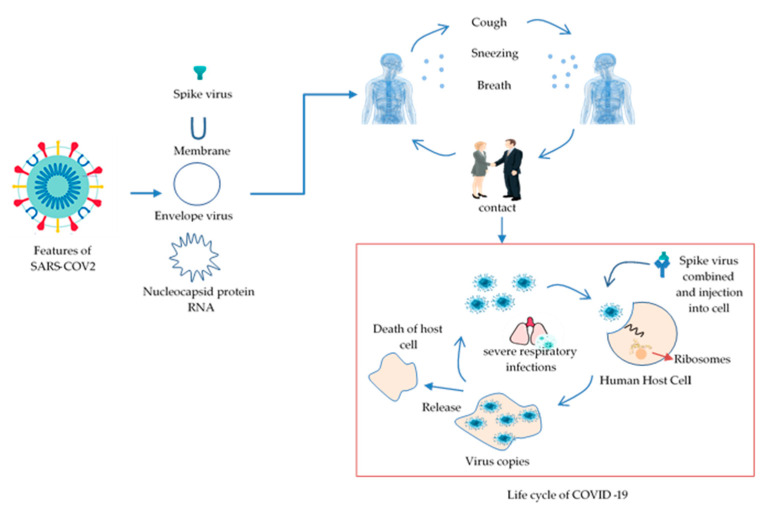
Illustration of the feature shape of COVID-19 and the transportation cycle to the lungs of humans.

This subsection begins by examining the emergence and features of COVID-19. Coronavirus families are a complex collection of viruses and can cause moderate to severe respiratory infections in humans and animals. Two types of zoonotic-coronaviral high pathogens, such as SARS and MERS viruses, occurred and caused a lethal respiratory disease in humans in 2002 and 2012 and became a new public health issue in the 21st century. Evolving coronaviruses became a significant concern of the public health community [13,14,15,16]. In late December 2019, several health facilities in Wuhan, Hubei Province, China, reported clusters of patients with pneumonia of unknown cause [17]. Coronavirus groups are classified into four genera: Alpha-coronavirus, Beta-coronavirus, Gamma-coronavirus, and Delta-coronavirus [18]. The virus comprises four distinct components: a spike (S), a membrane (M), an envelope (E), and a nucleocapsid (N). It has the largest genome (26.4–31.7 kb) among all known RNA viruses. Additionally, the virus’s genetic material includes single-stranded RNA [19,20,21]. Figure 1 shows the shape of COVID-19 and the transportation cycle to the lungs of humans.

### 1.2. Background

Optics and photonics technologies are essential to diagnosing viruses because photonic sensors have many advantages. In particular, they have high-performance abilities such as high sensitivities, low cost, low power consumption, and ultra-low limits of detection. Moreover, photonics integration, microfluidics, and microelectronics on a single chip provide an attractive technical foundation for implementing lab-on-a-chip systems, which are critical for performing quick, multiplexed, and real-time experiments and a variety of application sectors [22]. Recent studies have explored developing sensors based on photonics such as visible-light laser diodes, infrared analytics arrays, narrowband optical filters, surface plasmonic resonance (SPR), surface-enhanced Raman scattering (SERS), fluorescence, and other laser techniques, in order to solve many of the challenges of biological detection [23,24,25,26]. A study showed optical biosensors based on a combined Mach–Zehnder interferometer. The methodology featured applying compatible microfluidics (COMS) to diagnose DNA without labeling [27,28,29,30,31]. Previous studies have reported the detection and classification of specific viruses via quantitative analysis and sorting of viruses and other particles in the micron and nanoparticle size ranges using light scattering and fluorescence measurements. Light detection and ranging (LiDAR) is a technology used for several long-range, low-concentration biological diagnoses in the environment. With this method, when a laser pulse is transmitted, the reflected signal will be detected using a UV xenon fluoride excimer laser source [32]. It is used to identify and characterize biogenic materials, for example, pathogenic bacteria in both laboratory and field environments [33]. It uses a quantum cascade laser (QCL) technique to identify polluted surfaces. The wavelength tuned was between 9.1 and 9.8 nm [34,35]. Erik and colleagues used infrared spectroscopy methods based on Fourier transform infrared (FTIR) and QCL in biological sensing and surface contaminants [36]. An approach based on surface-enhanced Raman scattering (SERS) was reported in the literature to build optical sensors for influenza virus detection [37]. SERS is used for identifying biomolecules such as bacteria, cancer cells, and cellular processes. It is also used to determine whether a person has consumed contaminated food [38]. A research team implemented a sensor platform connected with a smartphone, such as fluorescence microscopes using mobile data, to diagnose the flu virus [39]. Research demonstrated the detection of COVID-19 virus in saliva using the Raman spectroscopy analysis tool, with an accuracy of 91.6%, a sensitivity of 92.5%, and a specificity of 88.8% [40]. Femtosecond pulse lasers emitting near-infrared have been used to inactivate many viruses with no damage to human cells [41,42]. The development of a new sensor based on field-effect transistors (FET) for diagnosing COVID-19 infection in clinical samples has been described. Graphene sheets have been coated with an antibody specific for the SARS-CoV-2 spike antigen. The detection limit was set between 1 and 100 fg/mL [43]. The author used a microcavity structure based on photonics technology to fight COVID-19 [44]. A study showed that the implementation of virus lasers is similar to an analytical platform for biological diagnosis without investigating immobilization or multiple wash stages [45]. Quick and reliable diagnosis of individual virus particles in clinical samples based on the laser spectroscopic method was reported [46]. Among the various types of techniques, Raman spectroscopy is an advantageous technique for studying the shapes of viral proteins and nucleic acids and the assembly mechanisms and architectures of native viruses [47]. Other than that, a study reported a new technique for quickly visualizing the droplets emitted while speaking and different masks’ efficiency in reducing their spread. The methodology used a cheap visible laser to image particles passing through scattering light [48]. Furthermore, a study showed that Raman scattering microscopy can be used to detect COVID-19 quickly and without using any labeling [49].

## 2. COVID-19 Identification via Photonics

Viruses are the smallest molecules of pathogens known. However, most of them cause significant deterioration of human health. This section discusses the essential parameters that contribute to improved COVID-19 diagnosis by photonics on surfaces such as a laser spectrum with molecules and identifies parameters of a laser device to enhance detection of viruses.

### 2.1. Laser Spectrum with Molecules

**Figure 2:**
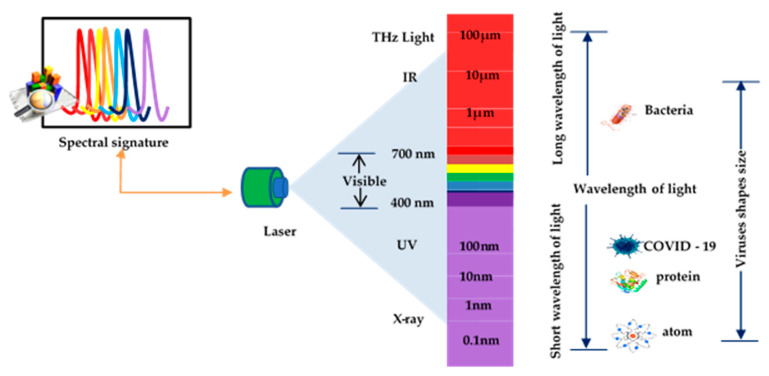
Comparison of the size of viruses for selecting a wavelength suitable to be used to diagnose COVID-19.

There are many viruses in the environment such as bacteria, fungi, pollen, and animal and plant debris. The smallest of all particles are viruses. They range in size from 10 to 300 nm. In contrast, red blood cells average about 6–8 microns, bacteria range from 1 to 4 microns, and fungi range from 5 to 10 microns. Spectroscopy signatures of laser properties can be used to identify single virus structural features that can be quickly determined. However, they cannot understand the biological structures because the light is often insensitive/blind to the distribution pattern of the constituent molecules due to a mismatch in wavelength scales between the virus size and wavelength of the laser. Consequently, this limits possible tracks to a qualitative change in portable detection instruments suitable for point-of-care testing [50]. Interactions between light and matter make a significant contribution in many scientific areas, creating critical spectroscopy applications, sensing, quantum information extraction, and lasers. Light waves spread at the speed of light in a vacuum, for most of these purposes. Several studies have explored the effects of combining novel laser beam techniques with molecules and have provided a wealth of new knowledge on the structure of atoms and molecules [41,42,45,51]. Light is considered electromagnetic waves and described as a stream of particles called photons. Each photon carries a ratio of energy. Matter can absorb photon energy, and spectroscopy is the science that analyzes the light emitted value. Light is classified according to its spectrum of wavelengths. When reacting with materials, it can cause ionization effects, according to the ascending order of wavelengths such as gamma rays, X-rays, ultraviolet rays, visible light, infrared rays, and microwaves. Light can be described in terms of waveforms with physical characteristics such as amplitude and wavelength. Correspondingly, the electromagnetic properties of light are that light has short waves with high energy and long waves with lower power. However, it can induce different phenomena when it interacts with matter because of the electromagnetic properties of light. Otherwise, when light of 100 nm wavelength interacts with a substance, it will cause the atoms to ionize. At the same time, the visible light (400 to 700 nm) wavelength is used to image targets in light microscopes and sometimes to visualize viruses because they lack enough magnification. It can detect a high percentage of viruses such as Mimivirus, Pithovirus, Megavirus, and Pandoravirus [52]. Figure 2 shows a comparison of the size of viruses for selecting a wavelength suitable to be used to diagnose COVID-19.

When light is incident on an atom, three necessary processes happen: absorption, spontaneous emission, and stimulated emission by which light interacts with particles. Depending on the type of matter, absorption occurs while the atom is of a lower energy level. The electron atom lifts it to the upper energy level by consuming the energy of the photon, creating up- and down-transitions between energy levels that are called spontaneous emission. The electron will be at an upper energy level (E2) or excited state and spontaneously decays to a lower energy level (E1) and radiates a photon light. The emitted photons contain energies peculiar to that substance, as each material has a particular collection of energy levels. The frequency (v) and wavelength (λ) of the light are related to these photon energies by

(1) $$\Delta Ε=\;\mathrm{hv}=\frac{hc}{\lambda }$$

where h is the Planck constant and c is the speed of light. The effectiveness of the laser application tool in the COVID-19 battle has many applications such as remote monitoring, tele-screening/telehealth, intelligent networks and big data, improving environmental quality, and improving the quality of foods.

### 2.2. Enhanced Detection of Viruses by Laser

In this section, the parameters of a laser device are identified and the essential keys for the detection of viruses are described. The size of a particle can be measured by observing spontaneous variations in the amount of light scattering. Therefore, it was utilized to study nanoparticles, such as counting the size of nanogold, molecules, gelatin, and crystalline substances. The laser system is classified according to the following aspects: lasing (active medium), wavelength, and temporal behavior. Amplification occurs in the lasing active medium in the state of solid, liquid, or gas. As a result, the lasers are classified as solid-state, liquid, or gas lasers. Among the essential medical lasers are CO_2_ (carbon dioxide), excimer (e.g., XeF), and Ar (argon) lasers. Liquid lasers include dye lasers, while solid-state lasers include ruby or Nd:YAG lasers. Lasers can also emit radiation in the ultraviolet, visible, and infrared (UV, visible, and infrared) parts of the electromagnetic spectrum. The three types are UV lasers, visible lasers, and infrared lasers. The temporal behavior in continuous-wave (CW) lasers and pulsed lasers has also been described. Some pulsed lasers produce high-speed pulses (femtoseconds); others have long pulses (milliseconds) [53]. The intensity Isc of the scattered light can be described as the following function of five variables:

(2) Isc=Isc(c,d,θ,λ,n)

According to the above equation, c is the concentration, d is the particle diameter, θ is the measurement angle, λ is the wavelength of light, and n is the refractive index of the particles compared to the surrounding medium scattering theory. Light scattering variables include wavelength, particle size, refractive index, incident angle, and concentration [54], as shown in Table 1.

## 3. Detection of COVID-19 Using Light Technologies

**Figure 3:**
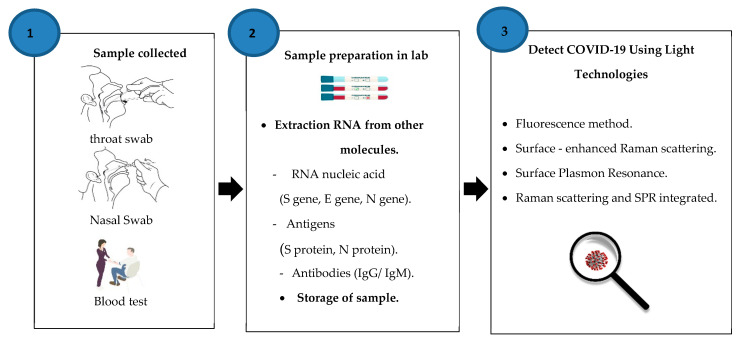
Steps to detect COVID-19 using light methods.

Our study focused on reliable sources in the literature review and examined and summarized 12 empirical studies of COVID-19 detection using laser techniques. This category includes research on photonics-based methods and applications for COVID-19 identification. Figure 3 shows the collected sample methods from patients, sample preparation, sample storage, and extraction RNA before use of laser techniques such as fluorescence methods, surface-enhanced Raman scattering spectra, surface plasmon resonance (SPR), and Raman scattering with SPR integrated. More importantly, we highlight the detection of COVID-19 based on photonics methods and the practical techniques suitable for reducing the spread of the epidemic.

### 3.1. Fluorescence Methods

Fluorescence spectroscopy with powerful emitters is a unique technique for identification and imaging down to the single molecule size, which is attributed to its ultra-high sensitivity. It is similarly named metal-enhanced fluorescence or plasmon-enhanced fluorescence. It uses a plasmonic nanomaterial to produce a fluorophore phenomenon (typically metals). It is achieved by putting the fluorophore near a metallic nanostructure, which causes the fluorophore electrons to connect to the resulting local plasmonic electric field. Hence, it would be exposed to a more powerful electric field, leading to enhanced emission and increased fluorescence enthusiasm [55,56,57,58]. This method employs fluorescence techniques to determine the presence of COVID-19 RNA. The method uses roughly 734 tests, including 593 throat and 141 sputum swabs from 670 patients at various hospitals. The technique reached 100 percent sensitivities and 99 percent specificities with both forms of sampling [59]. Detection of the SARS coronavirus protein in human serum samples was achieved using a fiber-optic biosensor based on localized surface plasmon coupled fluorescence (LSPCF) at the limit of detection 1 pg/mL [60]. Researchers recently developed laser-induced fluorescence-light detection and ranging (LIF-LiDAR) combined to detect and classify emerging virus strains such as Zika, Ebola, and COVID-19 in an environment. The method uses a laser multiwavelength excitation and receiving system which collects the imitated signals such as scattered light and fluorescence from the laser-interrogated target [61]. However, the threshold for the fluorescence signal is obtained by the standard divergence of cycles 3–15 of the average baseline fluorescence. While the cycle threshold (CT) is established by the number of PCR cycles needed to report quantifiable fluorescence, fluorescence is greater than the threshold of the fluorescence signal. As a result, a lower CT value implies there is a higher viral RNA load. CT 40 is generally clinically positive for the diagnosis of COVID-19 infection. The China CDC recommends a value of 37, which indicates that a suspect is clinically positive, and a value above 40 is considered clinically negative. Furthermore, the steps in the process are sample collection, storage, transportation, filtration, and treatment.

### 3.2. Surface-Enhanced Raman Scattering

Raman scattering is a mechanism in which a sample’s physical and chemical properties are determined by the recognizable spectral signatures associated with them, including light–molecule interactions. Spectroscopy is used for studying and analyzing the light reflected from molecules due to contact and has significantly proven to be highly sensitive and effective for diagnostics [62,63,64]. It has a number of advantages, including high selectivity due to the possibility of a special fingerprint signature, no signal interference from the analyte medium, single molecule detection, the ability to conduct multiplex sensing with a single laser beam, a high throughput, and applicability using commercially available portable Raman detectors [65]. A diagnostic of COVID-19 was developed based on surface-enhanced Raman scattering (SERS) combined with microfluidic systems that involve connected microchannels conjugated with Au/Ag-coated carbon nanotubes. The tool is used to detect viruses from various biological fluids, such as saliva, nasopharyngeal secretions, and tears [66]. Research demonstrated that the SERS technique could detect COVID-19 in polluted water at the single virus level. The detection limit was 80 copies ml^−1^, and the detection time was 5 min [67]. However, the SERS methods used to detect COVID-19 need sample collection, storage, transportation, filtration, and treatment before diagnosis, which are time-consuming. Moreover, there are the problems of a weak signal and low sensitivity with a low protein concentration.

### 3.3. Surface Plasmon Resonance (SPR)

SPR is an optical sensing technique used to diagnose biomolecular interactions in real time. The photon energy excitation needs a coupling medium through the interface. It can investigate using shape structures of an optical system, for example, prism coupling (a technique known as the Kretschmann design), localized surface plasmon (LSPR), waveguide, fiber-optic structures, and grating structures [68,69,70,71,72,73], and is used to monitor and target single molecules [55,74]. The resonance state necessary to accomplish SPR is as shown in Equation (3):

(3) $$\sqrt{\varepsilon_{p}}\sin\theta_{res}=\sqrt{\frac{\varepsilon_{m}\;\varepsilon_{d}}{\varepsilon_{m}+\varepsilon_{d}}}$$

where $\varepsilon_{p}$, $\varepsilon_{m}$, and $\varepsilon_{d}$ denote the dielectric constants of the substrate (prism, optical fiber backbone, etc.), a plasmonic material (metals), and a dielectric layer (analyte medium), respectively, and $\theta_{res}$ denotes the incident resonance angle. According to the equation, variations in the optical properties of the metal plate and the dielectric/sensing layer affect the depth, the direction (angle or wavelength), and the phase of the observed SPR change. According to published studies, optical biosensors such as SPR and LSPR have been extensively used in lab conditions to identify virus strains, such as those associated with SARS and MERS, and have been established commercially since the early 1990s [75]. SPR was used to measure and characterize the kinetics of severe acute respiratory syndrome (SARS). A study evaluated the kinetics of SARS binding to RNA during the phosphorylation of the SARS nucleoprotein (N protein) after it appeared in 2002–2003 [76]. In terms of plasmonic sensors, there are a few reports of COVID-19 detection using a variety of methods, for example, LSPR–sidelong flow [77], the LSPR–DNA selection approach [78], and the LSPR–PCR model. Additional information about the different identification strategies is available elsewhere [79]. Interestingly, developing an optical sensor LSPR for viral RNA samples, the authors of one study designed an alternate research approach utilizing a biosensor. For stability, the sensor mixed two different effects: optical and thermal. The biosensor artificially created DNA receptor sequences complementing the RNA genome parts of COVID-19 based on nanoparticle gold constructs on a glass substratum. These unique sequences were grafted onto the gold nanoparticles, detecting COVID-19 reliably. The team warned, however, that further improvement was required before application. As a result, the experiment achieved a high sensitivity at a lower detection limit of 0.22 pM [80]. The fiber-optic absorbance platform (P-FAB) biosensor-based SPR with a gold nanoparticle coating was developed to identify COVID-19 without pre-processing the patient’s saliva sample [81]. An experiment to create an optical sensor dependent on evanescent wave absorbance (EWA) for quick and precise COVID-19 diagnosis was reported at the point of care. The strategy is based on two concepts. The first objective was to assess the host’s immune response, and the second objective was to detect viral cell surface proteins using appropriate receptors. On the other hand, the host immune response was not a good indicator of the current virus, and other coronavirus pathogens such as SARS and MERS could cause similar reactions [82]. However, to develop SPR, a multi-stage process involves designing, manufacturing, functionalizing, and characterizing processes. The choice of the materials forming the reactive and inert regions of the biosensor is a vital aspect of the design since their variety is responsible mainly for functional selectivity. By examining the following factors, the functionalization technique may be determined as a function of the composing elements: such as adequate coverage of the metallic surface (plasmonic area) and accurate alignment of the active biomolecules, selectivity between the inert and the reactive regions, the geometry between the two functionalization methods, appreciation and specificity of the recognition event between both the analyte and the molecules, and time with cost.

### 3.4. Raman Scattering with SPR Integrated

**Figure 4:**
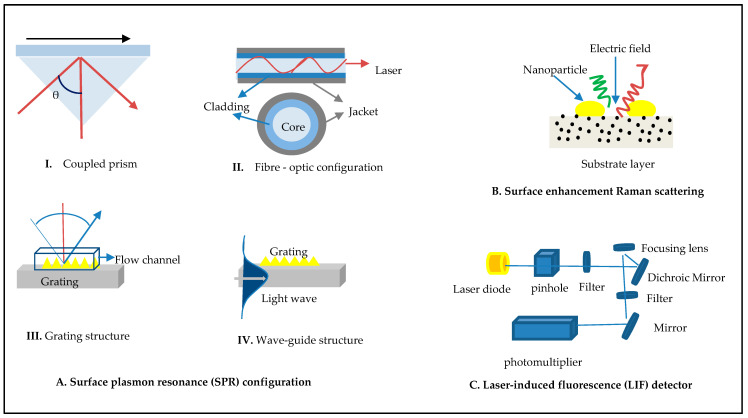
Illustration of light technologies’ structures: (A) surface plasmon resonance (SPR) configuration, (B) surface enhancement Raman scattering, and (C) laser-induced fluorescence (LIF) detector.

Raman scattering is a technique that is similar to fluorescence. Both processes begin with the absorption of a photon and finish with the emission of a dispersed or fluorescent photon, though there are significant limitations. Middle East respiratory syndrome (MERS) has been detected using a multiplex SERS platform based on plasmonic LSPR. The method employed was to deposit silver nanoparticles on 3D cellulose paper in order to produce a large number of inter-particles for enhancing the Raman signal [83]. E. Kim and colleagues introduced a new optical method, called multiplex SERS detection, based on fluorophore separation from the post-PCR requirements without modifying the primer and probe sequences and plasmonic sheet for detection capability for COVID-19. It uses silver nanodots on a plasmonic sheet to enhance the sensitiveness of the biosensor based on LSPR [84]. SERS-PCR had a lower detection limit than real-time PCR. These findings shed light on the possibility of reducing PCR cycles by the use of highly sensitive SERS. We agree that this technology is novel and can grow into a highly effective method for fast, responsive, and precise diagnosis. However, Raman signals are often weak, can only characterize materials in their solid-state, and have trouble detecting analytes in liquids. Additionally, detection of COVID-19 requires collecting samples from patients, isolation, and washing before detection, and it also consumes time. Therefore, it is important to consider the following factors while detecting analytes with SERS techniques: nanostructure geometry, substrate design, viral sizes, wavelength, hot spot generation, laser type, and power [85]. Table 2 shows an outcome analysis of studies on the diagnosis of coronaviruses based on laser techniques. Figure 4 presents the following details: part (A) shows plasmonic surface resonance structures such as traditional plasmonic based on a coupled prism, plasmonic based on the grating (long/short) period, and plasmonic based on a waveguide; part (B) presents surface-enhanced Raman scattering using nonpractical materials such as gold and graphene to improve the sensitivity of viruses; and part (C) shows structures of laser-induced fluorescence. The relationship between the wavelength of the laser and reflective index (RI) is nonlinear. Thus, changing the refractive index can reduce the velocity wavelength of the laser in the optic structure. It is also important to manage the quantity of light reflected when reaching the interface and the critical angle for total internal reflection.

## 4. Analysis Outcome of Literature Review

Coronavirus disease (COVID-19) quickly spreads, threatens human health, and has significant economic and social implications globally. Infectious agents possess specific receptor enzymes that enable them to attach to the host cell. They penetrate the circulation through our lungs, triggering a pathogenetic cascade in which they disrupt the immune system, resulting in cough, cold, fever, lung infection, organ failure, and even death. Reverse transcription-quantitative polymerase chain reaction (RT-qPCR) tools are used to evaluate patients with COVID-19 after collection of virus RNA isolated from the throat or nasopharyngeal swabs. Although the assays’ high specificity and theoretical sensitivity are approved for COVID-19 molecular detection, false negatives appear to be reported at a very high rate. In addition, the detection limit, known as the lower limit of detection or LOD (limit of detection), is the smallest amount of material that can be detected from its absence. It is sometimes confused with sensitivity. However, there is an error (false negative) in the sample measurement, indicating that it may include impurities with the concentration of the collected sample [86]. The most common methods of COVID-19 diagnosis require several hours and are uncomfortable; samples are collected by swabbing the nose or throat. Thus, the COVID-19 pandemic has shown the challenges of most existing analytical technologies for biological detection. Due to a lack of capabilities for quantifying or identifying the virus, the valid number of infected individuals and the actual fatality rate of patients infected with COVID-19 remain unknown [87]. However, emphasis on developing new, improved diagnosis methods is needed. Photonics provides powerful techniques for detecting viruses because the laser’s properties can classify and identify viruses according to wavelength emissions such as a spectrum signature. In addition, it is possible to collect big data from any probe laser through a fiber-optic network to enhance healthcare quality. However, to diagnose a virus, you must know more precise information about its form and genetic material.

Generally, the identity of a pathogen can be determined either by determining its genetic material or by detecting its unique markers. Most techniques depend on targets to a diagnosis of COVID-19 such as RNA nucleic acid (S gene, E gene, N gene, RNA-dependent RNA polymers), antigens (S protein, N protein, nucleocapsid N), antibodies (IgG/IgM), and the whole virus. However, it is molecularly diagnosed mainly by the discovery of virus RNA. While identification by viral proteins is possible, unlike nucleic acids, proteins cannot be amplified directly; therefore, direct detection of trace amounts of viral proteins is complex and may have some detection limitations [21].

However, ideal optical techniques for the detection of COVID-19 should exhibit many essential characteristics such as high sensitivity, accuracy (molecular specificity), fast (seconds or minutes, not hours and days), label-free, mass manufacturing, adaptability, portability (airports, hospitals, etc.), and cost-effective for global use [88].

## 5. Opportunities and Limitations

Several detection techniques for diagnosing viral infections have been established depending on the type of virus and its properties and the sample obtained from infected patients. Currently, research has focused on recognizing genetic material (DNA, RNA) and antibodies from clinical serum tests to diagnose COVID-19 rather than detecting the shape feature for the virus on the surfaces [66]. False negative outcomes from actual COVID-19 patients may have negative consequences, including delayed treatment for critically ill patients and a high chance of asymptomatic transmission. The World Health Organization has listed many distinct explanations for false negative findings [9]. The virus load in a tissue varies according to the time of infection and the collected place. For instance, the viral load of nose and mouth swabs varies according to the collection date following the onset of symptoms. Viral titers in the upper respiratory tract are higher early in infection but decrease with time. The complex viral loads present at various locations in the disease confuse specimen selection, resulting in false negative findings [89,90]. Although many technologies for identifying COVID-19 particles are viable, many problems limit their potential purpose. Moreover, the additional problem is that challenges include the following:

♦ Reduced performance of sensitivity and accuracy;
♦ Time-consuming preparation and purification of samples;
♦ The devices’ complex process;
♦ A need for highly skilled professional staff.

As a result, we need more effective methods to identify COVID-19 rapidly. Implementation of these techniques must be in a way that ensures increased precision, the efficiency of service and flexibility, and wide-ranging availability to assess the broad public. This study has evaluated our modern knowledge of the various optical biosensor techniques used to identify viruses and the potential rapid diagnosis of more people to contain the spread of this virus. Figure 3 shows an overview of sample collection, preparation, storage, and extraction steps before diagnosis and challenges of laser techniques. The issues are provided in Table 3, and the challenges of laser diagnosis techniques of COVID-19 are explained.

## 6. Tracking COVID-19 Virus in the Environment

Up to now, most techniques have some common properties for the diagnosis of COVID-19. Over the last four decades, photonics has brought many developments and inventions through experimental laboratory and theoretical research of a wide range of lasers. One of the most common models is a fiber laser based on erbium-doped fiber amplifiers (EDFA). A new methodology has been described using a semiconductor laser, which depends on a flow cytometer with expanded light scatter sensitivity for examining nanosized viruses [91]. The development of plasmonic fiber-optic sensors for the diagnosis of COVID-19 has potential by combining other laser techniques such as SERS and fluorescence imaging to detect temperature, blood, and nasal swabs of COVID-19 patients. Photonics biosensing technologies are promising for quickly identifying harmful viruses such as COVID-19 and provide unique techniques for controlling fresh virus outbreaks due to their high accuracy, low cost, ease of application, and ready-to-use mode [7,20,92,93,94]. As a result, the physical properties of the fiber waveguide configuration have brought forth a realistic opportunity for many applications. Optical transmission is used nearly everywhere in the communications sector. This can involve everything such as mobile phone calls and internet access points. Optical transmission is a significant area that improves healthcare appliances and reliability on a large scale. Fiber lasers are used to process data efficiently and reliably, are stable and scalable, and are a highly accurate tool in the diagnostics of viruses. For this reason, photonics technology can be used to achieve remote monitoring of COVID-19 propagation through a multi-sensor intelligent network and contribute to diagnosing COVID-19 in foods, point of care, tests of surface cleanliness, assessing the temperature of a person, ECG traces, and probe lasers to detect the virus directly from the nose or mouth of people at a low intensity. From another perspective, using a 3D scanning laser in reverse engineering systems to transform physical components to digital data has been popular in the medical field for scanning the human body and its features in precise dimensions. Handheld 3D scanners have ushered in significant changes in the healthcare sector, becoming a critical step in treating various ailments. Portable 3D scanning is essential for multiple healthcare uses, such as designing and fabricating personalized prosthetic and orthotic systems that may accommodate the patient’s unique anatomy [95]. Three-dimensional scanning technology studies a patient’s body shape, skin, tissue kinetic energy, chest, and individual body parts, among other things. As medical evidence varies between patients, the generation of interactive 3D model constructs and 3D digital images using computer projections combined with virtual reality can improve medical treatment [96,97]. It is also a helpful tool for remotely diagnosing and measuring viruses in the environment and assisting emergency teams. These data can be used for various purposes, including 3D scanning for virtual thoracic reality, motion control, autonomous imaging, and industrial design.

**Figure 5:**
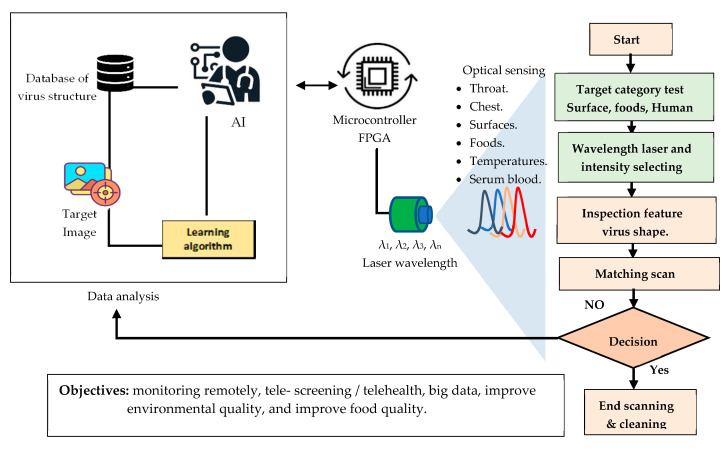
Framework illustrating detection of COVID-19 in the environment.

Finally, optical technologies can detect and control virus outbreaks through tests on surfaces/the environment and diagnose human infections in vitro. Our proposed system for tracking and detecting COVID-19 on surfaces based on AI will help medical teams by offering real-time remote monitoring and detection, increasing the efficiency and accuracy of optical biosensors, and enhancing health safety in public spaces such as universities, supermarkets, and ports, as well as test food quality, as shown in Figure 5.

## 7. Conclusions and Prospects

Researchers have come a long way in the fight against COVID-19. However, a new variant of the virus could threaten progress. Human exchange and touching surfaces are more risky ways to spread viruses. For this reason, we need rapid and accurate tools for the detection of viruses to reduce epidemic outbreaks. This study provided an overview of COVID-19 and the understanding of the detection of coronaviruses using light technologies. According to the data we have collected, photonics technology is considered fast, accurate, and practical to identify viruses. In this work, we proposed integrating lasers with artificial intelligence through a micro-controller to predict, in any image, captured with accuracy and speed, the viruses present on surfaces. In summary, laser-assisted identification using artificial intelligence algorithms can be helpful for pollution protection, virus density analysis in the air, and food safety inspection. Thus, data for virus detection can be gathered from an intelligent optical network of medical biosensors, thereby enhancing healthcare quality.

## Figures and Tables

**Table 1:** Keys of light scattering to enhance diagnosis of viruses.

Parameters of Laser Device	Description
Wavelength (λ)	The intensity of the scattered light reduces as the wavelength increases (inversely proportional). This outcome depends on the particle size, and for small particles, it is more pronounced.
Particle size (d)	Scattered light strength is highly dependent on the size of the particles.
Refractive index (n)	The scattering intensity is directly proportional to the difference in the particle’s refractive index and the medium. The smaller the difference, the lower the scattering intensity.
Scattering angle (θ)	The scattering intensity depends on the light angle of the incident.
Concentration (c)	The intensity of the scattered light is proportional to the size of the concentration.

**Table 2:** Analysis of studies in the literature on the detection of coronavirus disease (COVID-19) based on laser techniques.

Photonics Technique	Target of Virus	Material Coating	Limit of Detection	Wavelength	Diagnosis of COVID-19		Time Duration	Ref.
					In Clinical	On Surfaces		
LSPCF	SARS/nucleocapsid protein	Graphene sheet	0.1 pg/mL	658 nm	✓	✕	10 min	[60]
Fluorescence	COVID-19 RNA	Gold	1000 TU mL^−1^	NA	✓	✕	2–3 h	[59]
LIF-LiDAR	COVID-19, Zika, Ebola	NA	9.59 × 10^4^ PFU/cm^2^	266–550 nm	✓	✕	NA	[61]
SERS microfluid	COVID-19	Au/Ag	NA	NA	✓	✕	~few min	[66]
SERS	COVID-19	Gold nanoparticles	17.7 pM	785 nm	✓	✕	5 min	[67]
SPR	SARS/ N-protein	Quantum dots	0.1 pg mL^−1^	345 nm	✓	✕	1 h	[75]
SPR	Coronavirus/ N-protein	NA	2.17 nM	214 nm	✓	✕	20 min	[76]
LSPR	COVID-19/ spike protein	AuNIs	0.22 ± 0.08 pM	532 nm	✓	✕	800 s	[80]
P-FAB	COVID-19/ antibody IgM and IgG	AuNP	106 particles/mL	520–545 nm	✓	✕	15 min	[81]
EWA-LSPR	COVID-19/ antibody IgM and IgG	Gold nanoparticle	37 pM	LED	✓	✕	1 h	[82]
SERS-LSPR	MERS	Silver nanodot	1–106 nM	500 to 800 nm	✓	✕	NA	[83]
SERS-LSPR	COVID-19	Silver nanodot	153.53, 230.37 pM	526 nm 558 nm	✓	✕	More 2 h	[84]

**Table 3:** The challenges of laser diagnosis techniques of COVID-19.

Laser Diagnosis Techniques	Limitations
Fluorescence method	Collection of sample consumes time. Low sample size. Fluorophore has a short lifetime. Interference is possible.
Surface-enhanced Raman scattering	Weak signal relative to background. Low sensitivity with low protein concentration. Laser wavelength is unstable. Consumes time to collect sample. Noise signal interference.
Surface plasmon resonance	Low selectivity. A small perception depth. Mass transport challenge. Heterogeneity of surface. Misinterpretation of data. Collection of sample is time-consuming.
Raman scattering with SPR integrated	Collection of sample consumes time. Weak signal. At high analyte concentrations, there is failure to identify to virus, and identification is nonlinear. Nonuniform absorption of the molecules onto the nanoparticle surface leads to a decrease in signal intensity.

## Data Availability

Not applicable.

## Abbreviations

COVID-19	Coronavirus disease
SARS-CoV-2	Coronavirus severe acute respiratory syndrome 2
LSPCF	Localized surface plasmon coupled fluorescence
SERS	Surface-enhanced Raman scattering
SPR	Surface plasmon resonance
LSPR	Localized surface plasmon resonance
P-FAB	Plasmonic fiber-optic absorbance biosensor
EWA	Evanescent wave absorbance
pg	Picogram
ng	Nanogram
LIF-LiDAR	Laser-induced fluorescence-light detection and ranging
PCR	Polymerase chain reaction
WHO	World Health Organization
SARS	Severe acute respiratory syndrome
MERS	Middle East respiratory syndrome
CDC	Centers for Disease Control and Prevention
RU	Response unit
